# MitoCLox: A Novel Mitochondria-Targeted Fluorescent Probe for Tracing Lipid Peroxidation

**DOI:** 10.1155/2019/9710208

**Published:** 2019-11-13

**Authors:** Konstantin G. Lyamzaev, Natalia V. Sumbatyan, Alexey M. Nesterenko, Ekaterina G. Kholina, Natalia Voskoboynikova, Heinz-Jürgen Steinhoff, Armen Y. Mulkidjanian, Boris V. Chernyak

**Affiliations:** ^1^Belozersky Institute of Physico-Chemical Biology, Lomonosov Moscow State University, Moscow, Russia; ^2^Department of Chemistry, Lomonosov Moscow State University, Moscow, Russia; ^3^M.M. Shemyakin and Yu.A. Ovchinnikov Institute of Bioorganic Chemistry Russian Academy of Sciences, Moscow, Russia; ^4^Department of Biology, Lomonosov Moscow State University, Moscow, Russia; ^5^Department of Physics, University of Osnabrueck, D-49069 Osnabrueck, Germany; ^6^Department of Bioengineering and Bioinformatics, Lomonosov Moscow State University, Moscow, Russia

## Abstract

Peroxidation of cardiolipin (CL) in the inner mitochondrial membrane plays a key role in the development of various pathologies and, probably, aging. The four fatty acid tails of CL are usually polyunsaturated, which makes CL particularly sensitive to peroxidation. Peroxidation of CL is involved in the initiation of apoptosis, as well as in some other important cellular signaling chains. However, the studies of CL peroxidation are strongly limited by the lack of methods for its tracing in living cells. We have synthesized a new mitochondria-targeted fluorescent probe sensitive to lipid peroxidation (dubbed MitoCLox), where the BODIPY fluorophore, carrying a diene-containing moiety (as in the C11-BODIPY (581/591) probe), is conjugated with a triphenylphosphonium cation (TPP^+^) via a long flexible linker that contains two amide bonds. The oxidation of MitoCLox could be measured either as a decrease of absorbance at 588 nm or as an increase of fluorescence in the ratiometric mode at 520/590 nm (emission). In CL-containing liposomes, MitoCLox oxidation was induced by cytochrome c and developed in parallel with cardiolipin oxidation. TPP^+^-based mitochondria-targeted antioxidant SkQ1, in its reduced form, inhibited oxidation of MitoCLox concurrently with the peroxidation of cardiolipin. Molecular dynamic simulations of MitoCLox in a cardiolipin-containing membrane showed affinity of positively charged MitoCLox to negatively charged CL molecules; the oxidizable diene moiety of MitoCLox resided on the same depth as the cardiolipin lipid peroxides. We suggest that MitoCLox could be used for monitoring CL oxidation in vivo and, owing to its flexible linker, also serve as a platform for producing peroxidation sensors with affinity to particular lipids.

## 1. Introduction

Mitochondria, as sources of reactive oxygen species (ROS), are major targets of oxidative damage. The inner mitochondrial membrane (IMM) is particularly sensitive to lipid peroxidation (LPO) since it harbors both ROS-producing respiratory enzymes and easily oxidizable lipids such as cardiolipin. The specific peroxidation of cardiolipin, which is exclusively located in the inner mitochondrial membrane, could play an important role in the regulation of some intracellular signaling pathways and in apoptosis [1–3]. The excessive LPO in the inner mitochondrial membrane could significantly contribute to various pathologies and to aging. Therefore, the prevention of LPO by mitochondria-targeted, membranophilic antioxidants has high therapeutic and antiaging potency [4–6].

Studies of cardiolipin peroxidation are hindered by the absence of means to trace it in real time. Currently, such an analysis requires chromatographic and mass spectrometric techniques [7]. Our goal is to obtain a cardiolipin-specific fluorescent LPO probe(s). Such probes should be able to accumulate in mitochondria and have a specific affinity to CL. Earlier, Prime and colleagues developed a mitochondria-targeted fluorescent LPO probe, MitoPerOx [8]. In this probe, the BODIPY(581/591) moiety was conjugated with the membrane-penetrating triphenylphosphonium (TPP) cation by propionyl aminoethyl linker [8]. Generally, TPP-carrying compounds selectively accumulate in mitochondria being driven by the mitochondrial membrane potential (160–180 mV, negative inside) [9]. During the last decades, TPP^+^ moieties were applied to deliver diverse antioxidants and fluorescent probes into mitochondria [5, 10, 11]. MitoPerOx was shown to accumulate in mitochondria and to report lipid peroxidation [8].

We also relayed on the widely used fluorescent LPO probe C11-BODIPY (581/591) that consists of a BODIPY fluorophore bearing a conjugated diene moiety sensitive to peroxidation and a C11-hydrocarbon tail as a membrane anchor [12]. The oxidation of the diene results in a strong increase in the fluorescence emission at 520 nm whereas the initial fluorescence at 590 nm decreases. These properties allow the application of C11-BODIPY (581/591) as a ratiometric dye. In addition, we were encouraged by data of Birk and colleagues who have shown that the SS-20 peptide (Phe-*D*-Arg-Phe-Lys-NH_2_) could enter mitochondria and selectively target CL [13]. Because CL is the major negatively charged phospholipid in the mitochondrial membrane, a chimera of MitoPerOx and SS20 with several positive charges could show a specific affinity to cardiolipin and report its oxidation. Indeed, it was shown that TPP-containing compounds effectively displaced molecules of N-nonyl acridine orange (NAO), a specific cardiolipin probe, from mitochondrial membranes [14]. The affinity to cardiolipin might contribute to the effective prevention of cardiolipin oxidation by TPP-containing quinolic antioxidants and presumably underlay the high efficiency of TPP-based mitochondria-targeted antioxidants in various physiological models [2].

Here, we describe the synthesis and characterization of a new mitochondria-targeted lipid peroxidation probe MitoCLox where a BODIPY (581/591) fluorophore was conjugated with TPP residue using a long flexible linker that contained two peptide bonds to imitate the SS-20 peptide. This linker is currently used by us as a scaffold for inserting additional positive charges. MitoCLox reported the cytochrome c-dependent oxidation of CL in liposomes and did not interfere with the TPP-based mitochondria-targeted antioxidant SkQ1. Molecular dynamic simulations showed that the diene residue of MitoCLox was localized within the lipid bilayer next to the cardiolipin lipid peroxides and therefore should be efficiently oxidized by lipid radicals. The *in vivo* application of MitoCLox for the analysis of mitochondrial LPO in living cells is presented in a separate article (Lyamzaev et al. “The Novel Fluorescent Mitochondria-Targeted Probe MitoCLox Reports Lipid-Mediated Response to Oxidative Stress.” *Oxid Med Cell Longev*, 2019, this issue).

## 2. Materials and Methods

### 2.1. Chemical Synthesis of MitoCLox

**Figure 1:**
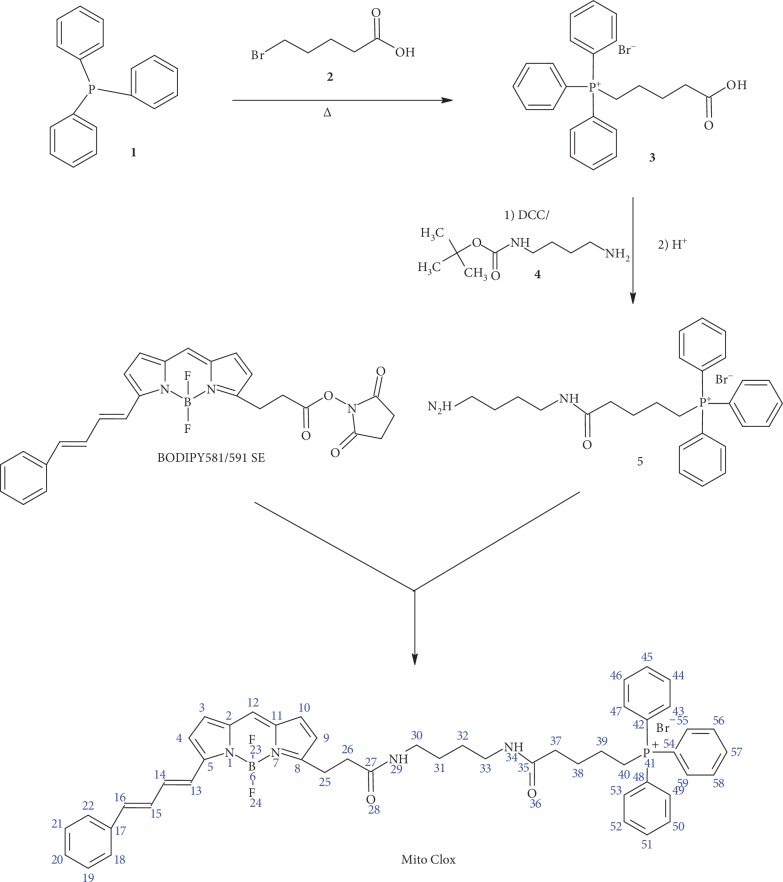
Scheme of chemical synthesis of MitoCLox.

MitoCLox was synthesized from 4-difluoro-5-(4-phenyl-1,3-butadienyl)-4-bora-3a,4a-diaza-s-indacene-3-propionic acid succinimidyl ester (BODIPY581/591 SE) (Invitrogen Life Technologies) and {5-[(4-aminobutyl)аmino]-5-oxopentyl}(triphenyl)phosphonium bromide (5) (Figure 1).

Initially, triphenylphosphine (TPP, 1) (1.0 g, 3.8 mmol) was condensed with 5-bromovaleric acid (2) (688 mg, 3.8 mmol) for 12 h at 85^о^С. The product (4-carboxybutyl)(triphenyl)phosphonium bromide (3) was purified on silica gel 60 using the chloroform-methanol mixture (4 : 1 (*v*/*v*)) as eluent to give the pure product with 90% yield (1.51 g). TLC: *R_f_* (chloroform-MeOH, 4 : 1) 0.30; LC-MS: *τ*(HPLC) = 0.78 min(ACQUITY BEH C18 (2.1 × 50 mm, 1.7 *μ*m) column (Waters); 0.5 ml/min, 20 mM formic acid, gradient 5-100% MeCN for 3 min); *m*/*z* calculated for C_23_H_24_O_2_P^+^, 363.2, found 363.2.

The product 3 was introduced (108 mg, 0.25 mmol) into the reaction with *tert*-butyl (4-aminobutyl)carbamate (4) (47 mg, 0.25 mmol) in the presence of 1.5 equivalents of N,N-dicyclohexylcarbodiimide (DCC, 78 mg, 0.375 mmol) and 2 equivalents of diisopropylethylamine (DIPEA, 0.085 ml, 0.5 mmol) in dichloromethane (1 ml, a small amount of DMF was added until completely dissolved) for 2 h under cooling and 12 h at room temperature. The reaction mixture was diluted with 20 ml of dichloromethane and 10 ml of 0.1 M aqueous HCl. Aqueous layer was extracted with dichloromethane (2 × 10 ml); combined organic layers were washed with water (2 × 5 ml), 5% solution of NaHCO_3_ (1 × 5 ml), water (2 × 5 ml), and saturated NaCl (1 × 3 ml) and dried over anhydrous Na_2_SO_4_; and the volatiles were evaporated *in vacuo*. The residue was crystalized from the MeOH-diethyl ether mixture, and the product (tert-butyloxycarbonyl derivative of compound 5) was isolated from the precipitate on silica gel column eluting with solvent system dichloromethane: MeOH, 7 : 1. Yield: 100 mg (65%) TLC: *R_f_* (chloroform–MeOH, 4 : 1) 0.70; *R_f_* (chloroform-MeOH-water, 65 : 25 : 4) 0.65; LC-MS: *τ*(HPLC) = 1.06 min (ACQUITY BEH C18 (2.1 × 50 mm, 1.7 *μ*m) column (Waters); 0.5 ml/min, 20 mM formic acid, gradient 5-100% MeCN for 3 min);*m*/*z*calculated for C_32_H_42_N_2_O_3_P^+^, 533.29, found 533.2. To 27 mg (0.044 mmol) of the product 0.5 ml of 98% formic acid was added. The mixture was stirred for 6 h at room temperature and then evaporated *in vacuo*. The residue was dissolved in a minimal amount of methanol and treated with ether. The resulted precipitate was centrifuged and dried *in vacuo* to give the pure compound 5 as a thick colorless oil with 88% yield (20 mg). TLC: *R_f_* (chloroform-MeOH-25% aqueous NH_3_, 65 : 25 : 4) 0.10, *R_f_* (chloroform-MeOH-water-ethyl acetate-25% aqueous NH_3_, 336 : 130 : 17 : 5 : 10) 0.17; *R_f_* (chloroform-MeOH-water, 65 : 25 : 4) 0.30; LC-MS: *τ*(HPLC) = 0.91 min (ACQUITY BEH C18 (2.1 × 50 mm, 1.7 *μ*m) column; 0.5 ml/min, 20 mM formic acid, gradient 5-100% MeCN for 3 min); *m*/*z* calculated for C_27_H_34_N_2_OP+, 433.2, found 433.4.

For the synthesis of MitoCLox, a solution of of compound 5 (3.2 mg, 6.15 *μ*mol) in sodium bicarbonate buffer (0.1 N, pH 9.2, 0.2 ml) was added to the solution of BODIPY581/591 SE (2.0 mg, 4.1 *μ*mol) in N-methylpyrrolidone (0.2 ml). The mixture was kept for 2 h at room temperature in the dark, and 0.1 M HCl was added to adjust pH 7.0. The product was extracted with dichloromethane (2 × 3 ml), washed with water (2 × 1 ml), dried, evaporated in vacuo, and separated on a silica gel by using a chloroform-methanol mixture (7 : 1 (*v*/*v*)) as an eluent. The pure product triphenyl(5-{[4-(4,4-difluoro-5-(4-phenyl-1,3-butadienyl)-4-bora-3a,4a-diaza-s-indacene-3-(propionylamino)butyl]amino}-5-oxopentyl)phosphonium chloride (MitoCLox) was obtained as a blue solid with 80% yield (2.8 mg). In some cases, additional purification was performed using a C18 column (10 × 250 mm, 5 *μ*m bead size) at a 5 ml/min flow rate in 20-80% gradient of acetonitrile in 0.01% aqueous TFA for 20 min (*τ* = 16.5 min). TLC: *R_f_* (chloroform-MeOH, 4 : 1) 0.65; *R_f_* (chloroform-MeOH, 7 : 1) 0.29; UV (MeOH):*λ*_max_ = 585 nm, 347 nm, 334 nm; fluorescence (EtOH): *λ*_ex_ = 585 nm, *λ*_em_ = 595 nm; LC-MS: *τ*(HPLC) = 2.27 min (ACQUITY BEH C18 (2.1 × 50 mm, 1.7 *μ*m) column; 0.5 ml/min, 20 mM formic acid, gradient 5-100% MeCN for 3 min); *m*/*z* calculated for C_49_H_51_BF_2_N_4_O_2_P^+^, 807.38, found 807.59; ^1^H NMR (500.13 MHz, *CDCl_3_*) *δ* ppm 0.91 (m, 8H, 31, 32, 38, 39), 1.11-1.79 (m, 8H, 25, 22, 29, 34, 37), 3.19-3.79 (m, 6H, 30, 33, 40), 6.41-7.23 (m, 9H, 13-16, 14, 15, 19, 21, 22), 7.41 (m, 5H, 18-22), 7.78 (m, 15H, 43-47, 49-53, 55-59). ^13^C{^1^H}-JMOD NMR (125.76 MHz, *CDCl_3_*) *δ* ppm 29.51 (d, 1C, *J* = 165 Hz, 40), 29.71 (s, 4C, 31, 32, 38, 39), 31.94 (s, 1C, 25), 38.16 (s, 2C, 26, 37), 59.54 (s, 2C, 30, 33), 118.22 (d, 3C, *J* = 325 Hz, 42, 48, 54), 124.81 (s, 2C, 5, 8), 125.46-130.02 (m, 9C, 3, 4, 9, 10, 12, 13-16), 126.15 (s, 1C, 17), 128.20 (s, 2C, 2, 11), 130.79 (m, 6C, 43, 47, 49, 53, 55, 59), 134.30 (m, 3C, 45, 51, 57), 135.27 (m, 6C, 44, 46, 50, 52, 56, 58), 173.93 (bs, 2C, 27, 35).

### 2.2. Preparation of Cardiolipin Liposomes

The liposomes were produced by extrusion, based on the method of Hope and coworkers [15, 16], similar to our previous study [17]. Liposomes were prepared by suspending the CL from a bovine heart (Avanti Polar Lipids Inc., Alabaster, USA) as a powder at 3 mg/ml by 5 minutes of vortexing in a 50 mM sodium phosphate buffer (pH 7.4) containing 0.1 mM diethylenetriaminepentaacetic acid (buffer A) to bind possible traces of metals. Liposomes were obtained with a miniextruder equipped with two syringes (Avanti), each of 1 ml volume; a membrane with a pore diameter of 100 nm (Avanti) was used. The homogeneous lipid suspension was passed through the membrane 19 times. The liposome samples were stored on ice until use within the same day.

### 2.3. CL Oxidation in Liposomes

CL oxidation was initiated by cytochrome c (CytC) from an equine heart (Sigma-Aldrich, Cat. No. C2506). Liposomes in buffer A at a final CL concentration of 0.1 mM were incubated with CytC (1 *μ*M) at 37^o^C in a 3 mL quartz cuvette inside the UV-2450 spectrophotometer (Shimadzu, Tokyo, Japan) equipped with a Peltier thermoelement. Oxidation of CL was monitored by changes in absorbance at 234 nm which correspond to the formation of conjugated dienes [18, 19]. The value of the molar absorptivity of conjugated dienes was taken as 27400 M^−1^ cm^−1^. SkQ1 also absorbed in the UV range (with a maximum at 267 nm), and this absorbance decreased with reduction to SkQ1H_2_; however, the “oxidized minus reduced” spectra had an isosbestic point close to 234 nm and therefore did not contribute to the absorbance changes at 234 nm. Spectra were recorded every 5 minutes at the wavelengths of 210–600 nm and against a reference cuvette containing the same components, except for CytC. SkQ1, MitoCLox, and the other additions were done to both chambers either initially or 30 minutes after the start of the experiment.

To reduce SkQ1 to the corresponding quinol (SkQ1H_2_), 2-3 mg of dry sodium borohydride was added to 1-2 mM SkQ1 solution in ethanol. The excess of reductant was removed by a small volume of fuming HCl followed by at least two centrifugations at 15800 g (5 minutes each) to remove the sodium borate pellet. The reduced SkQ1H_2_ was stored at −80°C until use.

### 2.4. Molecular Dynamic Modeling

For molecular dynamic modeling of the membrane bilayer, we used phospholipid composition close to that of the inner mitochondrial membrane [20]: 1-palmitoyl-2-linoleoyl-phosphatidylcholine(PLPC), 1-stearoyl-2-linoleoyl-phosphatidylcholine (SLPE), 1-palmitoyl-2-oleoyl-phosphatidylcholine (POPC), and tetralinoleoyl cardiolipin-cardiolipin (TLCL) mixed in the ratio of 7 : 21 : 7 : 8. The initial structure was prepared with a CHARMM-GUI server [21]. We used the CHARMM36 force field for simulation [22]. The force field of the dye was obtained from the *ab initio* density functional theory (DFT) calculations that were carried out with the Firefly package [23] using the 6-31G∗ basis set and the B3LYP5 functional. The package performed energy minimization in delocalized coordinates built as linear combinations of internal coordinates, with the rotating angle constrained, whereby the decision on which vectors do form the optimization subspace could affect the calculated energies. Thus, we deposited the protocol as well as the resulting rotating movie over calculated coordinates at each step (see the XYZ movie file) in the Github archive (https://git.io/JevG6).

The partial charges derived from quantum chemical calculations were implemented into the force field. Additionally, we parametrized dihedral angles around the bonds between diene carbons (C13-C14, C14-C15, and C15-C16 in Figure 1) of the phenyldiene tail. We calculated the potential energy surface and fit dihedral parameters to reproduce it in molecular dynamic (MD) simulations. Production MD simulations with 2-fs step in an NPT ensemble were of about 1000 ns; the preceding equilibration MD simulations were of about 100 ns.

To simulate CL mono-hydroperoxides, we modeled 8 variants of the oxidized molecule. There are four variants of hydroperoxides of linoleic acid [24]:

1. 13-Hydroperoxy-(*E*,*E*)-octadeca-9,11-dienoic acid (“13EE”)
2. 13-Hydroperoxy-(*Z*,*E*)-octadeca-9,11-dienoic acid (“13ZE”)
3. 9-Hydroperoxy-(*E*,*E*)-octadeca-10,12-dienoic acid (“9EE”)
4. 9-Hydroperoxy-(*E*,*Z*)-octadeca-10,12-dienoic acid (“9EZ”)

CL can carry oxidized glycero-1-acid or glycero-2-acid making the total amount of mono-peroxidized CL variants equal to eight.

To supplement the CHARMM force field, we performed *ab initio* calculations of the following small molecules:

1. 7-Peroxy-(*E,E*)-nona-3,5-diene
2. 7-Peroxy-(*Z,E*)-nona-3,5-diene

In both molecules, we calculated partial charges and the potential energy profile around the dihedral angle C3-C4-C5-C6.

Using the derived molecules, we constructed two model membranes: with four variants of CL with the peroxide at the 9th position and with four variants with the peroxide at the 13th position.

The archives with MD simulation results are deposited at GitHub: https://github.com/comcon1/MitoMDMembranes.

## 3. Results and Discussion

### 3.1. Synthesis

MitoCLox was synthesized from 4-difluoro-5-(4-phenyl-1,3-butadienyl)-4-bora-3a,4a-diaza-s-indacene-3-propionic acid succinimidyl ester (BODIPY581/591 SE, Invitrogen Life Technologies) and {5-[(4-aminobutyl)аmino]-5-oxopentyl}(triphenyl)phosphonium bromide (Figure 1). MitoCLox was synthesized in water-organic mixture (N-methylpyrrolidone/0.1 N sodium bicarbonate, pH 9.2) for 2 h at room temperature with the 80% yield after purification on silica gel. The structure was confirmed with 1H and 13C NMR spectra.

**Figure 2:**
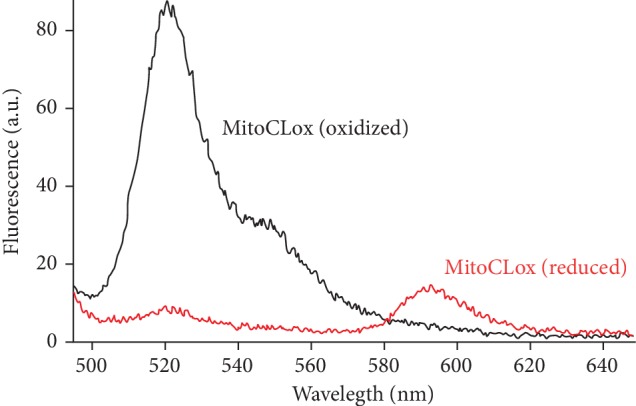
Fluorescence spectra of the oxidized (dark line) and reduced (red line) MitoCLox in CL-containing liposomes at 488 nm excitation. Oxidation of MitoCLox was induced by cytochrome c (for details, see Figure 3).

The fluorescence spectra of MitoCLox (reduced and oxidized forms) were measured in the liposomes (Figure 2) and did not differ from that of C11-BODIPY581/591 [8].

### 3.2. MitoCLox Oxidation in Cardiolipin Liposomes

**Figure 3:**
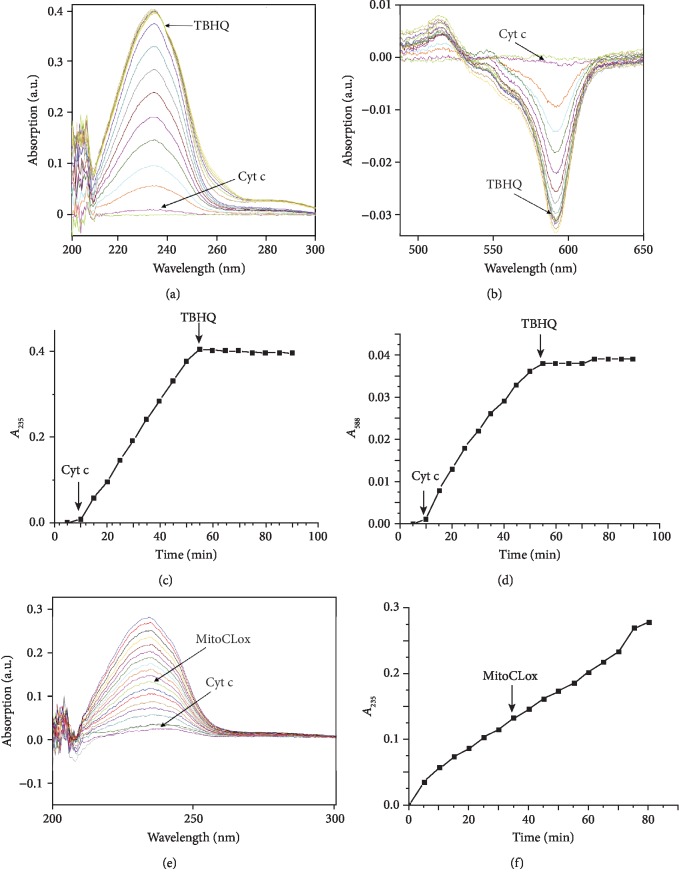
Cardiolipin oxidation in liposomes (100 *μ*M) as induced by the addition of 1 *μ*M cytochrome c. Additions are shown by arrows. (a, b) Absorbance spectra of liposomes around 235 nm (a, accumulation of diene conjugates) and around 595 nm (b, oxidation of MitoCLox); the arrow indicates the time of addition of the antioxidant tert-butylhydroquinone (TBHQ, 10 *μ*M). (c, d) Kinetics of accumulation of diene conjugates (c) and of the oxidized form of MitoCLox (d); the arrow indicates the time of addition of the antioxidant tert-butylhydroquinone (TBHQ, 10 *μ*M). (e, f) Effect of MitoCLox (300 nM, the addition is indicated by an arrow) on the rate of accumulation of dienes in cardiolipin liposomes upon stimulation of oxidation by cytochrome c (CytC, 1 *μ*M).

**Figure 4:**
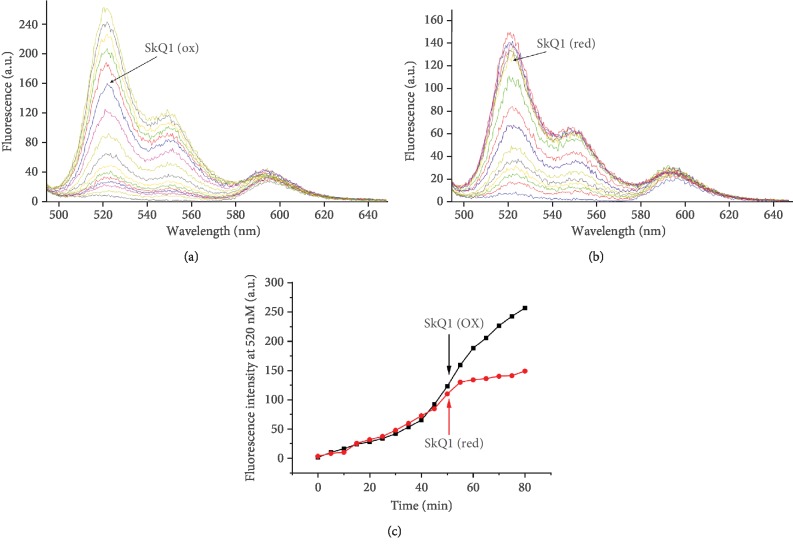
The effect of SkQ1 (1 *μ*M) in its oxidized (a) or reduced (b) form on the peroxidation rate of cardiolipin liposomes (100 *μ*M) as detected by MitoCLox (300 nM). (c) Kinetics of MitoCLox oxidation. The fluorescence of MitoCLox was measured with the excitation wavelength of 488 nm.

CL oxidation in liposomes was initiated by cytochrome c (CytC) and monitored via changes in absorbance at 234 nm which correspond to the formation of conjugated dienes [17, 25]. The oxidation of MitoCLox was registered either by a decrease of absorbance at 581 nm (Figure 3) or by changes in MitoCLox fluorescence (Figures 2 and 4). The absorbance measurements were instrumental because they allowed us to concurrently follow the oxidation of the dye and the accumulation of conjugated dienes. Changes in fluorescence spectra of MitoCLox did not differ from those of C11-BODIPY581/591 indicating that the products of the probe oxidation by lipid radicals are similar. Kinetics of MitoCLox absorbance changes, and kinetics of conjugated diene formation measured in the same assay were linear during at least 50 min after the addition of CytC (Figures 3(c) and 3(d)). In these experiments, approximately 20% of CL was oxidized. These data indicate that MitoCLox oxidation is proportional to CL oxidation in a wide range of oxidized CL/reduced CL ratios. These observations also indicate that the generation of reactive CL radicals in reaction with CytC is much slower than the formation of CL diene conjugates and oxidation of MitoCLox.

We applied MitoCLox for the analysis of various antioxidants in CL-containing liposomes. It was found that tert-butylhydroquinone (TBHQ) efficiently prevented the MitoCLox oxidation induced by CytC (Figures 3(a)–3(d)). After the addition of TBHQ, conjugated dienes did not form anymore (Figures 3(a) and 3(c)) and the absorbance of MitoCLox at 581 nm did not change (Figures 3(b) and 3(d)).

It is important that the addition of MitoCLox did not affect the oxidation of CL as was confirmed by monitoring the formation of conjugated dienes (Figures 3(e) and 3(f)). These data demonstrate that MitoCLox neither interfered with interaction of CytC with CL nor scavenged linoleic peroxy radicals in CL to a notable extent.

Figure 4 shows changes in the fluorescence of MitoCLox in a suspension of CL liposomes where peroxidation was induced by addition of CytC. Mitochondria-targeted antioxidant SkQ1 efficiently prevented the further MitoCLox oxidation induced by CytC (cf. Figures 4(a) and 4(b)). In the control experiments, the oxidized quinone form of SkQ1 did not affect the MitoCLox oxidation (Figure 4(c)). These data indicate that the cationic TPP residue of SkQ1 does not affect the interaction of MitoCLox with CL.

### 3.3. Molecular Dynamic Modeling

Dienes in phenyl-butadienyl residue of BODIPY581/591 are oxidized by chain-propagating species, especially derivatives of polyunsaturated fatty acids (PUFA) including LO^·^ and LOO^·^ [26]. Therefore, we studied the distribution of the dye in the model mitochondrial membrane by all-atom molecular dynamic simulation.

Ab initio potential energy surface (PES) scans of the MitoCLox molecule were performed using relaxed scan methodology implemented in Firefly v. 8+ as described in Materials and Methods. It is assumed that a relaxed surface scan gives lower energy values than a simple surface scan, and the resulting values are more close to the experimental ones.

**Figure 5:**
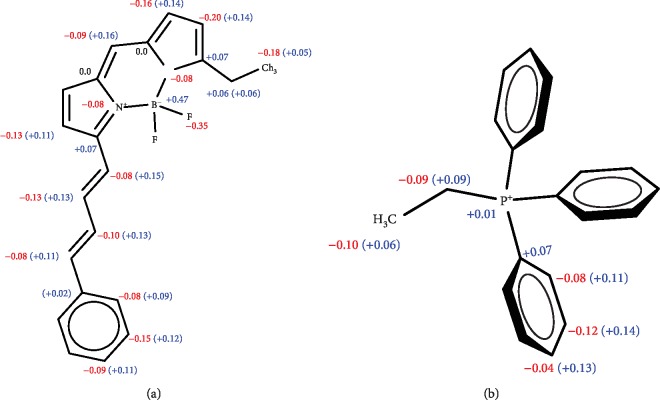
Partial charges of the molecular fragments calculated in order to develop the force field for the MitoCLox dye.

To create the mechanical model including new dihedral angles, we first calculated charges by the RESP procedure. We calculated the charges for the PDT-BODIPY part (a) and TPP part (b) of the molecule separately (Figure 5). Then, we fit the dihedral parameters to bring the MD potential energy profile close to the *ab initio* profile. The MD profile was verified by rotational script that is deposited at Github (https://git.io/JevGX). This script performs a relaxed scan in MD by the following steps: (1) fixing the angle of interest at some value, (2) performing energy minimization with the angle fixed, (3) performing the mechanical energy calculation by one-step dynamic run, and (4) repeating from step (1) with a new angle value.

**Figure 6:**
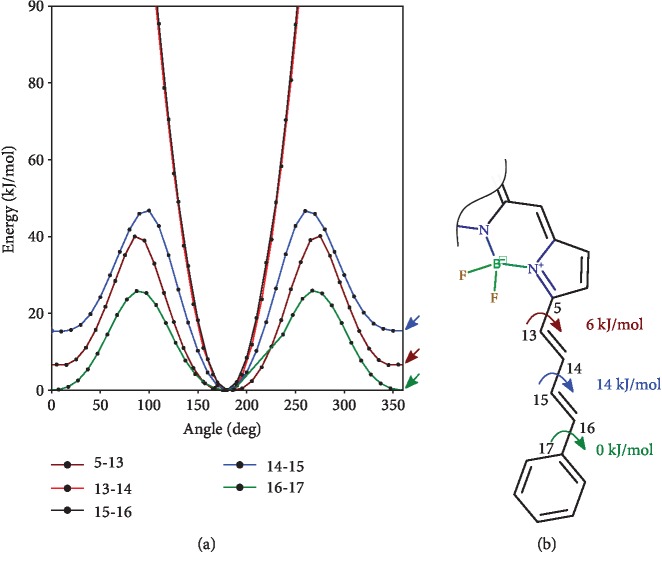
Potential energy surfaces (a) along the selected dihedral angles calculated as a relaxed scan with DFT (B3LYP5) using the 6-31G∗ basis set. The bond of rotation is indicated in the legend. The atom numbering is shown in (b) as well as cis-trans energy differences for single bonds (marked with arrows in (a)).

The only flexible part of the hydrophobic fragment is the butadienyl spacer between the BODIPY core and the phenyl group. Our *ab initio* calculations showed that some single bonds are relatively flexible (Figure 6) and that the dihedral angle around these bonds can change in the range of 50 degrees within the 2kT energy range. These *ab initio* results underlay our MD model of the dye; thus, the behavior of the classical model corresponded well to quantum estimations.

**Figure 7:**
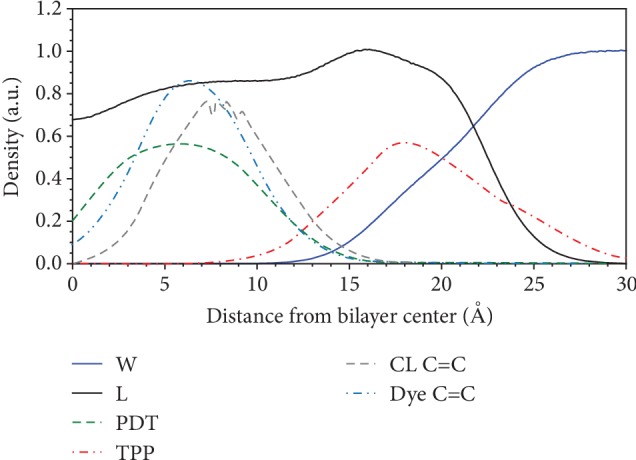
The density distributions of the following components at the bilayer-water interface: L: lipids; W: water; PDT: phenyl-butadienyl tail of the dye; TPP: TPP^+^; CL C=C: double bonded carbons of CL; dye C=C: diene carbons of the dye. The density is averaged over 700 ns part of the MD simulation trajectory of the MitoCLox-containing system. The density values are given in different units to be visually comparable: in g/cm^3^ (L, W), in 10 g/cm^3^ (CL C=C), and in 10 mol/cm^3^ (PDT, TPP, and dye C=C).

To analyze the mutual arrangement of MitoCLox and the surrounding lipids (especially CL), we simulated this dye in a bilayer of complex composition that resembled the inner mitochondrial membrane [20]. The model bilayer was composed of tetra-linoleoyl-cardiolipin (TLCL) and major zwitterionic phospholipids with choline and ethanolamine head groups (see Materials and Methods for details).  Figure 7 illustrates the density distribution of some system components along the normal to the bilayer plane. The hydrophobic phenyl-butadienyl side chain (green dashed curve) is located in the region of acyl lipid residues whereas the BODIPY core, the linker, and TPP^+^ (red dash-dotted curve) occupy the polar lipid head group region at the lipid/water interface. The depth density distribution of the diene carbons of MitoCLox (blue dashed curve) significantly overlapped with the distribution of TLCL double bonds (gray dashed line), which are the targets of peroxide radicals. The analysis of the density distribution of MitoCLox parts along the normal to the bilayer plane demonstrates that the dye is L-shaped. The phenyl-butadienyl side chain is oriented approximately along the normal to the bilayer plane while the linker dwells in the plane of the membrane. In agreement with this conclusion, the molecular dynamic modeling revealed the similar density distribution for MitoPerOx (with a shorter linker, see [8]) in the same membrane (not shown).

**Figure 8:**
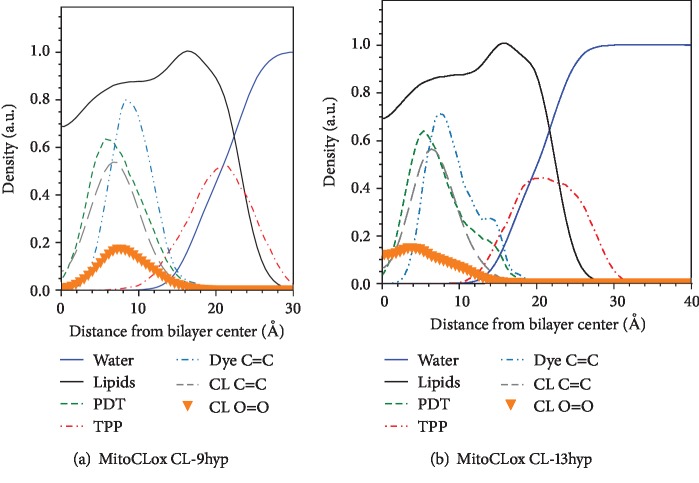
The density distribution of parts of the MitoCLox and CL peroxide groups in two oxidized bilayer models, namely, with the mono-9-hydroperoxy-CL (a) and mono-13-hydroperoxy-CL (b). Only the monolayer containing the dye is shown. The distributions of the same groups of MitoCLox as in Figure 6 are shown. The distributions of the peroxide group of oxidized CL (CL O-O) and double bonded carbons of CL (CL C=C) are also depicted. The density is averaged as in Figure 6.

Additionally, we checked the depth distribution of hydroperoxide moieties in the model of the oxidized membrane, where one linoleic acid in each CL molecule carried a hydroperoxide. We simulated separately the membranes containing CL with a hydroperoxide either at the 9th or at the 13th position (Figure 8). The depth distribution of the C9-hydroperoxide showed more overlap with the distribution of oxidizable diene in MitoCLox than that of the C13-hydroperoxide, which indicates that the hydroperoxide in the 9th position should be more efficient in oxidation of MitoCLox.

**Figure 9:**
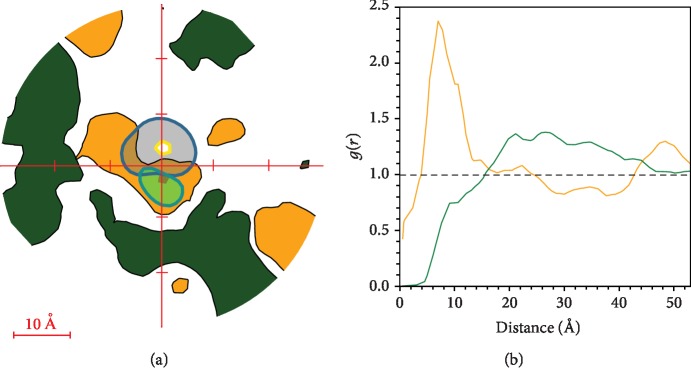
Distribution of TLCL and SLPE lipids in MitoCLox-containing monolayer. (a) All frames are aligned according to the plane projection of phenyldiene→TPP^+^ vector, which corresponds to the OY axis in the used coordinate system. For TLCL (orange) and SLPE (green), the areas with average concentration higher than 50% of the lipid concentration in the bilayer are plotted. Distributions of the phenyldiene tail (light green) and TPP (gray) are seen near the zero point. (b) Radial distribution function for dye-lipid distance measured between the phosphorus atom of TPP and C2 glycerol atom of TLCL (orange curve) or SLPE (green curve). This function was calculated over the trajectory of the MitoCLox simulation.

To study the interaction of the dye with different lipids, we analyzed the lateral distribution of CL and the most common phospholipid 1-stearoyl-2-linoleoyl-phosphatidylethanolamine (SLPE) in relation to the dye (Figure 9). Near the center of the top diagram, the distributions of the phenyl-butadienyl side chain (light green spot) and TPP (gray spot) are shown. We observed a highly populated area of CL (orange) near MitoCLox indicating a certain affinity of this dye to CL. For comparison, the areas highly populated with SLPE (dark green spots) are mostly located distantly from the dye indicating the absence of a specific affinity between SLPE and the dye. The same effect is clearly visible in plots of the radial distribution function of phosphorus atoms of TPP and the C_2_ glycerol atom of CL. This plot for MitoCLox shows a peak at ~25 Å for SLPE and a peak at ~7 Å for TLCL (Figure 9(b)). A very similar lateral distribution of MitoCLox in relation to CL and SLPE was also observed in the model of an oxidized bilayer (not shown).

**Figure 10:**
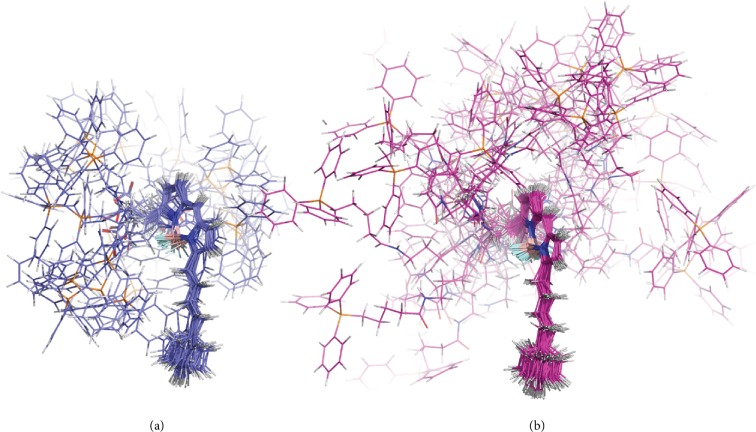
Flexibility of MitoPerOx (a) and MitoCLox (b) during the simulation in the model membrane. Frames are fitted by the BODIPY core and phenyldiene tail. Centroids of all clusters built with RMSD cutoff 0.3 are presented simultaneously.

Being relatively rigid in the BODIPY-butadienyl part, the structure of MitoCLox is much more flexible in the linker region in comparison with that of MitoPerOx (Figure 10). Such a high flexibility of the linker in the lipid bilayer might be important for interaction with CL molecules that are occluded within respiratory supercomplexes of the inner mitochondrial membrane [27].

## 4. Conclusions

Here, we show that MitoCLox reliably reports oxidation of CL in the membrane and, at the same time, does not quench the propagation of lipid radicals. The oxidation of MitoCLox could be prevented by mitochondria-targeted antioxidants concomitantly with the oxidation of CL. The observation that oxidized SkQ1 did not affect the oxidation of MitoCLox indicates that MitoCLox, in the *in vivo* experiments, could be combined with other TPP^+^-carrying fluorescent probes without danger of interference of these probes with the interaction between MitoCLox and CL.

The results of the MD modeling demonstrate that the TPP^+^ group of MitoCLox is located in the close proximity with the areas of CL high density in the bilayer. The results of MD simulations suggest that MitoCLox could reliably and, most likely, specifically react with CL radicals during membrane peroxidation. Higher flexibility of MitoCLox in comparison with MitoPerOx could enable its interaction even with those CL molecules that are occluded within respiratory supercomplexes [3, 25, 28–30].

## Data Availability

The web archives references for the data used to support the findings of this study are included within the article.
